# Uptake and Transport of Naringenin and Its Antioxidant Effects in Human Intestinal Epithelial Caco-2 Cells

**DOI:** 10.3389/fnut.2022.894117

**Published:** 2022-05-24

**Authors:** Zhen-Dong Zhang, Qi Tao, Zhe Qin, Xi-Wang Liu, Shi-Hong Li, Li-Xia Bai, Ya-Jun Yang, Jian-Yong Li

**Affiliations:** Key Lab of New Animal Drug Project of Gansu Province, Key Lab of Veterinary Pharmaceutical Development of Ministry of Agriculture and Rural Affairs, Lanzhou Institute of Husbandry and Pharmaceutical Sciences of CAAS, Lanzhou, China

**Keywords:** naringenin, uptake, transport, anti-oxidation, metabolomics

## Abstract

Naringenin, a flavanone, has been reported for a wide range of pharmacological activities. However, there are few reports on the absorption, transport and antioxidant effects of naringenin. The study was to explore the uptake, transport and antioxidant effects of naringenin *in vitro*. Cell transmembrane resistance, lucifer yellow transmission rate, and alkaline phosphatase activity were used to evaluate the successful construction of cell model. The results showed that the absorption and transport of naringenin by Caco-2 cells were time- and concentration-dependent. Different temperatures (37 and 4°C) had a significant effect on the uptake and transport of naringenin. Verapamil, potent inhibitor of P-glycoprotein, significantly inhibit naringenin transport in Caco-2 cells. The results revealed that naringenin was a moderately absorbed biological macromolecule and can penetrate Caco-2 cells, mainly mediated by the active transport pathway involved in P-glycoprotein. At the same time, naringenin pretreatment could significantly increase the viability of H_2_O_2_-induced Caco-2 cells. Twenty four differential metabolites were identified based on cellular metabolite analysis, mainly including alanine, aspartate and glutamate metabolism, histidine metabolism, taurine and hypotaurine metabolism, pyruvate metabolism, purine metabolism, arginine biosynthesis, citrate cycle, riboflavin metabolism, and D-glutamine and D-glutamate metabolism. We concluded that the transport of naringenin by Caco-2 cells is mainly involved in active transport mediated by P-glycoprotein and naringenin may play an important role in oxidative stress-induced intestinal diseases.

## Introduction

The gut is the primary site for nutrient absorption in all animals and humans (1). Many single traditional Chinese medicine (TCM), extracts of TCM, and some TCM monomers enter the blood through the intestinal tract after oral administration (2–4). Many parameters affect the intestinal absorption of substances and their bioavailability, such as transit and absorption time (5). The cell models *in vitro* have many advantages, such as good reproducibility, low cost, and short cycle. Therefore, research models *in vitro* are often used for drug transport and absorption studies. More and more cell models are used to explore the transport and absorption of flavonoids (6–8). Among them, Caco-2 cell model has a good correlation with *in vivo* research. It is also possible to explore the transit time and absorption capacity of the drug ingested from the AP side and the BL side. The transport and absorption mechanism of drug molecules via the intestines of humans will be further explored (9, 10). By calculating the Papp value, the absorption information at the cellular level can be determined (11).

Naringenin is a flavonoid compound that mainly found in grapes and oranges (12–14). Studies have shown that naringenin has anti-inflammatory, anti-oxidant, anti-diabetic, anti-cardiovascular disease, and anti-fibrosis pharmacological activities (15–20). Zhao.et al found that naringenin protects human umbilical vein endothelial cells from oxidative damage induced by palmitate via reducing autophagy flux (21). Li et al. also found that naringenin can stimulate skeletal muscle cells to take up glucose and increase insulin sensitivity *via* AMPK signaling pathway (14).

In this study, the model of Caco-2 cells was constructed. The transport, uptake, and antioxidant effects of naringenin in the monolayer culture model were investigated. This study provided evidence for the absorption, transport and antioxidant effects of naringenin in the gut.

## Materials and Methods

### Chemicals

Caco-2 cells were obtained from American Type Culture Collection (ATCC, Manassas, VA, USA). TranswellTM cell culture dish (12 mm membrane diameter) were obtained from Corning Costar Corp. (Cambridge, MA). Fetal bovine serum, MEM glucose medium, and cell culture flask were from Gibco (Grand Island, NY, USA). CCK-8 kit was obtained from Beyotime (Shanghai, China). Lucifer yellow (LY) was from Solarbio (Beijing, China). Alkaline Phosphatase Kit was from Mlbio (Shanghai, China). Gradeacetonitrile and formic acid (>99%, for LC-MS) were obtained from Thermo fisher scientific (Waltham, MA, USA).

### Cell Culture

Caco-2 cells were routinely maintained in 20% fetal bovine serum, 1% gluta-max, 1% sodium pyruvate, 1% non-essential amino acids, 77% modified eagle medium media supplemented at 37°C under humidified atmospheric conditions containing 5% CO_2_.

### Cell Viability

Cell viability was determined via CCK-8 assay (22).

### Establishment of Caco-2 Cells Model

**Figure 1:**
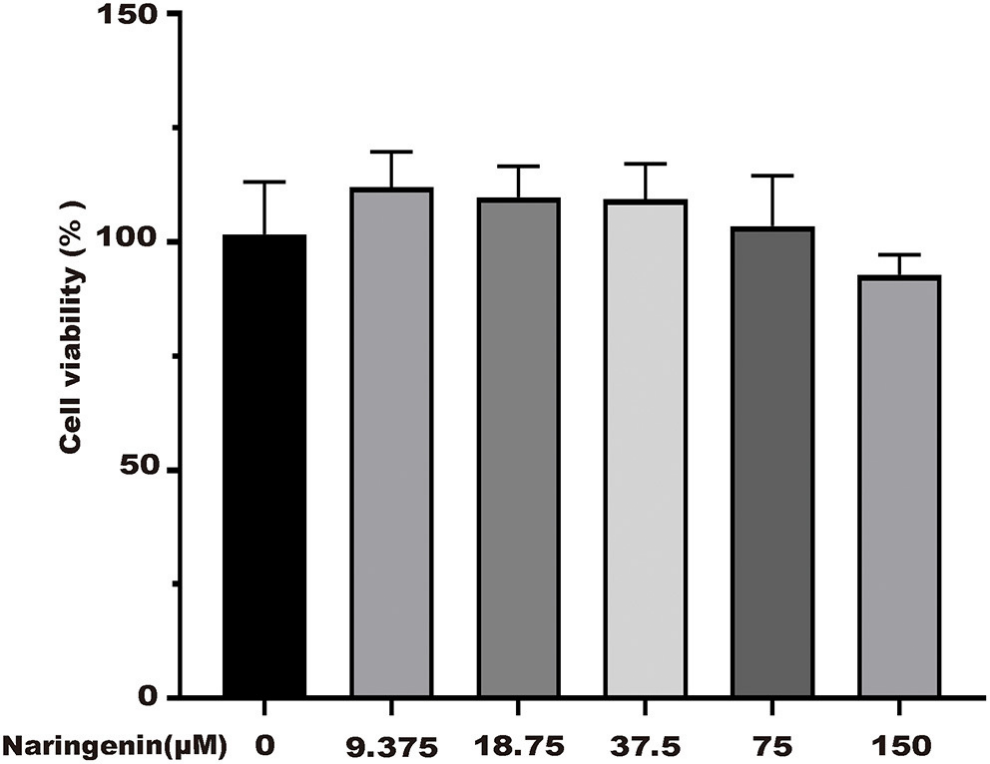
Effects of different concentrations of naringenin on cell viability. *p < 0.05 compared with control group.

**Figure 2:**
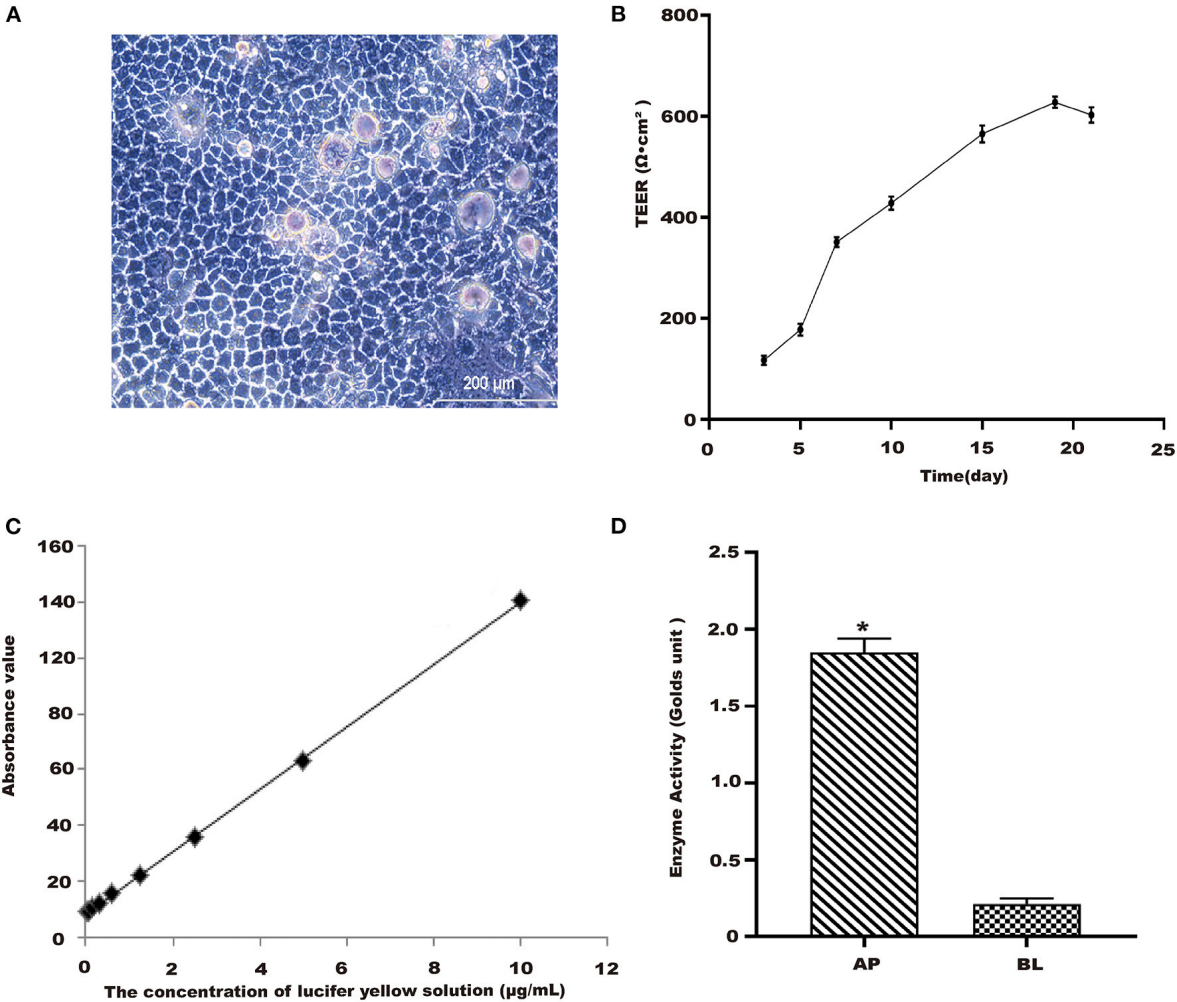
Establishment of Caco-2 cell model. **(A)** Morphology of Caco-2 cells observed in 10th day. Scale bar = 200 μm. **(B)** TEER of Caco-2 cells with time. **(C)** The standard curve of lucifer yellow. **(D)** Alkaline phosphatase activities. *p < 0.05 compared with BL side.

The Caco-2 cell model was established as previously published (23). Different concentrations of naringenin (9.375, 18.75, 37.5, 75 and 150 μM) had no effect on the activity of cells for 24 h (Figure 1). As shown in Figure 2A, when the Caco-2 cells were cultured for 21 days, the Caco-2 cells were tightly connected without gaps. From Figure 2B, the TEER value reached 602 Ω•cm^2^ on the 21st day. The results indicated that the monolayer membrane of Caco-2 cells had good integrity.

#### Lucifer Yellow Transmission Rate Experiment

After treatments, lucifer yellow transmission rate was assessed using the corresponding commercial kit according to the manufacturer's protocols (24). From Figure 2C, the linear regression equation of lucifer yellow absorbance was y = 0.0556x+0.0427 (R^2^ = 0.9997). The permeation rate of lucifer yellow with cells was determined as 0.42 (±0.15) × 10^−6^ cm•s^−1^, the well without cells was 3.9 (±0.01) × 10^−5^ cm/s. The results showed that Caco-2 cells were fully differentiated and morphologically intact after being cultured for 21 days.

#### Alkaline Phosphatase Activity Assay

Alkaline phosphatase kit was used to detect the enzyme activity of AP and BL side (23). It could be seen from Figure 2D that the alkaline phosphatase activity of the AP side was significantly higher than that on the BL side. This model could be used as an *in vitro* cellular model for subsequent transport and uptake experiments.

### Analytical Methods

#### Sample Extraction

In total 200 μL methanol was added to the 200 μL sample and centrifuged at 14,000 g for 10 min. The supernatant was collected and evaporated to dryness, reconstituted in methanol, and analyzed by HPLC.

#### Liquid Chromatography Analysis

Naringenin chromatographic analysis was performed on a Sursil ODS-B column (250 × 4.6 mm, 5 mm particle size). The mobile phase consisted of an acid solution containing a 0.2% phosphoric acid and methanol (40:60, *v/v*) at a flow rate of 0.4 mL/min. Naringenin elution was recorded at a constant wavelength of 282 nm.

### Transport Assay of Caco-2 Cells

Different concentrations of naringenin solutions (9.375, 18.75, 37.5, 75 and 150 μM) were added on the Caco-2 cells. In total 100 μL of sample solution was collected from the BL side at different times (15, 30, 45, 60, 90, and 120 min) and then 100 μL of HBSS was added.

### Caco-2 Cells Uptake Experiment

Different concentrations of naringenin solution (9.375, 18.75, 37.5, 75, and 150 μM) were added on the Caco-2 cells. The cells were collected at different time periods (15, 30, 45, 60, 90, and 120 min), respectively.

### Metabonomic Analysis

#### Cellular Metabolite Extraction

After incubation, the Caco-2 cells were collected. In total 80% ice methanol was added to the Caco-2 cells, incubated at low temperature for 5 min, and the Caco-2 cells were scraped from the cell culture plate. Samples were lysed by three freeze-thaw cycles and pelleted by centrifugation at 14,000 × g for 10 min at 4°C. Twenty μl of supernatant from each sample was taken and mixed well to prepare quality control (QC) samples. The samples were then filtrated through 0.2 μm filters into sample vials.

#### UHPLC-QE Orbitrap/MS/MS Conditions

LC-MS/MS analyses were performed using a HPLC system with a HSS T3 column coupled to Q Exactive (Orbitrap MS, Thermo). The mobile phase A was 0.1% formic acid in water and the mobile phase B was acetonitrile. The elution gradient was set as follows: 0 min, 2% B; 1 min, 2% B; 18 min, 100% B; 22 min, 100% B; 25 min, 2% B. The flow rate was 0.3 mL/min. The injection volume was 2 μL. The QE mass spectrometer was used for its ability to acquire MS/MS spectra on an information-dependent basis (IDA) during an LC/MS experiment. ESI source conditions were set as following: Aux gas flow rate as 16 Arb, Full ms resolution as 70,000, Collision energy as 25 eV in NCE model, MS/MS resolution as 17,500, and spray voltage as −3.0 kV (negative) or 3.6 kV (positive), respectively.

#### Qualitative Analysis of Metabolites

The raw data were converted into “Analysis Base File” (ABF) format files by ABF Converter software. The peak detection, deconvolution and peak alignment in data processing were performed using the MSDIAL 2.2.62 software. The data obtained are imported into SIMCA (version 14.1). We performed PCA and OPLS-DA on the data in SIMCA The Human Metabolome Database (HMDB) was used to search for accurate mass values of differential metabolites. Cluster analysis and pathway analysis of differential metabolites were performed using MetaboAnalyst 5.0.

### Statistical Analysis

SAS 9.2 (SAS Institute Inc., NC, USA) was used for statistical analysis. All data are presented as means ± SD. Statistical significance was considered at *p* <0.05.

## Results

### Transport Experiment Results

#### The Transport Results of Naringenin in Caco-2 Cells at Different Times

**Figure 3:**
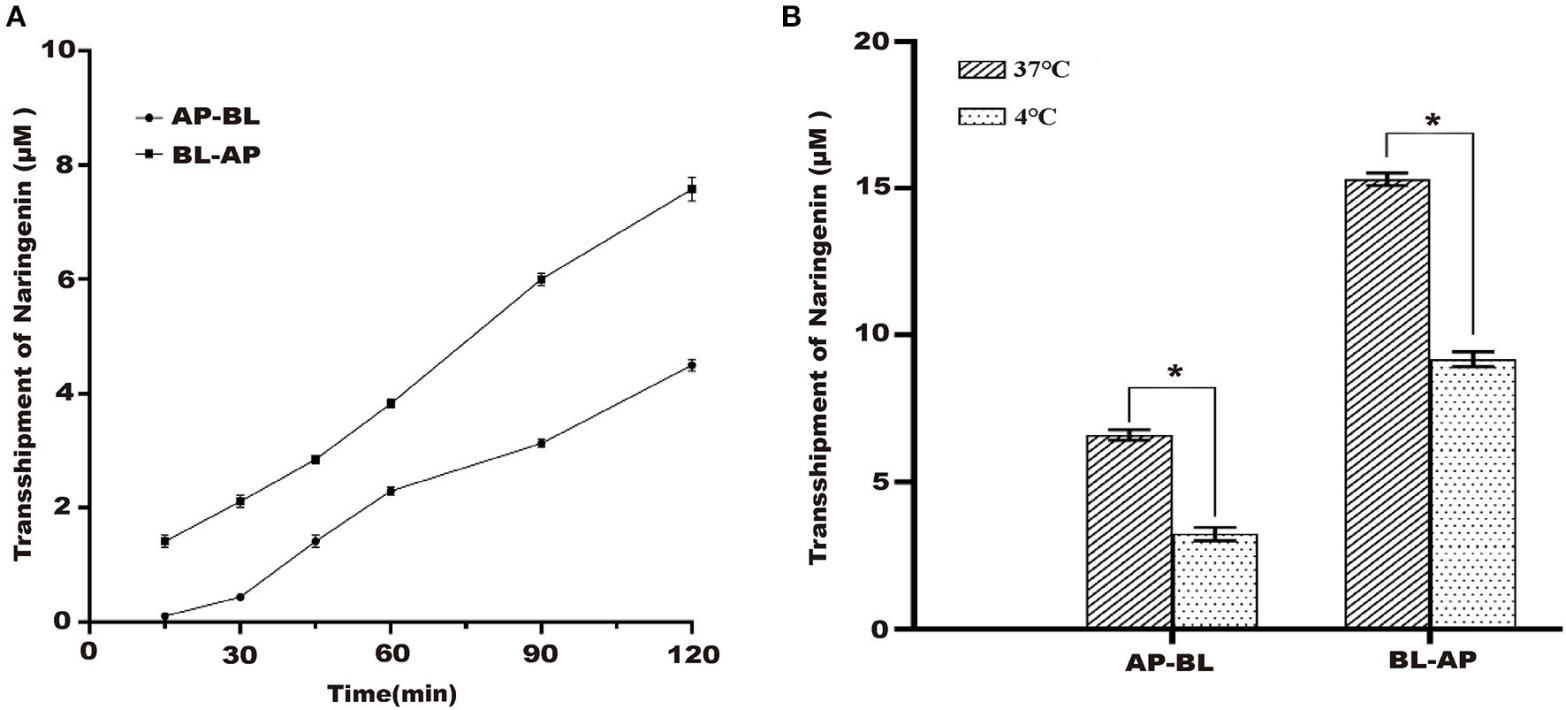
Effects of different times and different temperature on the transport of naringenin. **(A)** The effects of AP-BL and BL-AP on the transport of naringenin (75 μM) at different times. **(B)** Effects of different temperatures on naringenin transport. *p < 0.05 compared with 37°C.

Under the condition of 37°C, the naringenin transport volume on both sides of Caco-2 cells gradually increased with the increase of time, and the transport volume reached the maximum at 120 min but did not reach the saturation state (Figure 3A).

#### The Transport Results of Naringenin in Caco-2 Cells at Different Temperatures

37 and 4°C were selected to study the effect of temperature on the transport of naringenin. The results showed that compared to 37°C, the transport volume in Caco-2 cells was significantly reduced at 4°C. This indicated that temperature has a significant effect on its transport capacity (Figure 3B).

#### Transport of Naringenin on Caco-2 Cells

**Figure 4:**
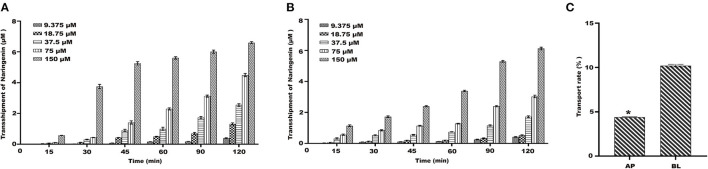
The transport of naringenin with different concentrations (9.375–150 μM). **(A)** The amount of naringenin transported from the AP side to the BL side. **(B)** The amount of naringenin transported from the BL side to the AP side. **(C)** The transport rate of naringenin Caco-2 cells. *p < 0.05 compared with BL side.

Under the condition of 37°C, the results showed that the transport amount of naringenin in Caco-2 cells gradually increased with the increase of the concentration (Figures 4A,B). The naringenin transport rate on the BL-AP side was 10.09%, and the naringenin transport rate on the AP-BL side was 4.89%, and the BL-AP side was significantly higher than the AP-BL side (Figure 4C).

### Papp of Transport of Naringenin

**Figure 5:**
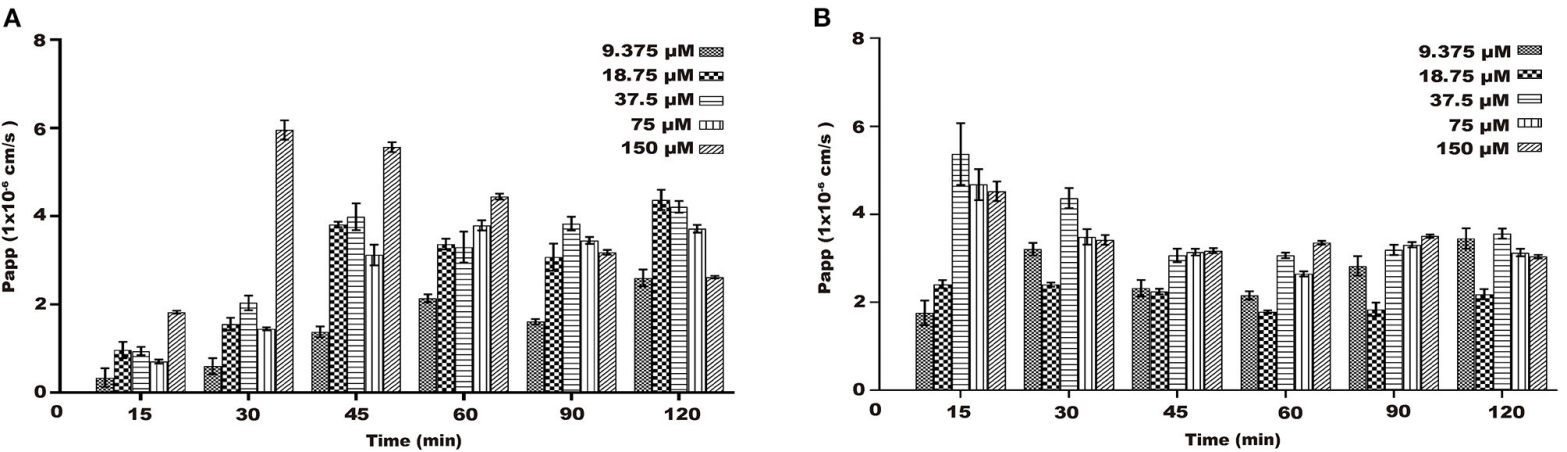
The Papp of each concentrations (9.375–150 μM) of naringenin during 120 min. **(A)** From AP side to BL side. **(B)** From BL side to AP side.

On the AP-BL side, the Papp values of naringenin at different concentrations increased with time and concentration during transport (Figures 5A,B). During the transportation of different concentrations of naringenin on the AP-BL side, the Papp value of 150 μM was the maximum value at 30 min. Interestingly, on the BL-AP side, the Papp values of different concentration groups gradually decreased with time. Similarly, the Papp values of the same concentration and different time groups also decreased gradually. The results indicated that there may be active transport of naringenin in Caco-2 cells.

### Uptake Result

#### The Absorption of Naringenin by Caco-2 Cells at Different Times

**Figure 6:**
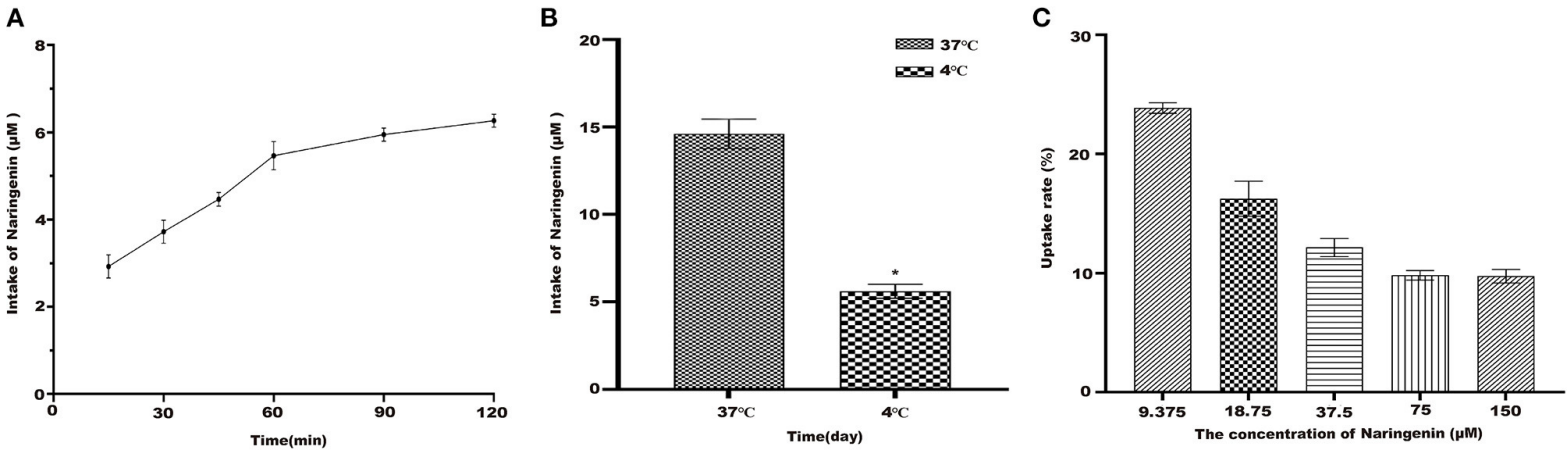
The uptake of naringenin with different concentrations (9.375–150 μM) of cells during 120 min. **(A)** The effect cells on the uptake of naringenin (75 μM) at different times. **(B)** The effect of temperature on uptake of naringenin. **(C)** The uptake rate of naringenin of Caco-2 cells. *p < 0.05 compared with 4°C.

Under the condition of 37°C, naringenin was constant in Caco-2 monolayer cells (Figure 6A). The results suggested that Caco-2 cells have a time-dependent uptake of naringenin.

#### The Absorption of Naringenin by Caco-2 at Different Temperatures

As shown in Figure 6B, compared with 37°C, the intake of naringenin at 4°C was significantly reduced. It was possible that low temperature affects the fluidity of cell membranes. As shown in Figure 6C under the condition of 37°C and 120 min, the uptake rate of naringenin by Caco-2 monolayer cells decreased with the increase of the concentration, and finally tended to be saturated.

### The Impact of Verapamil on Naringenin Tansport and Uptake

**Figure 7:**
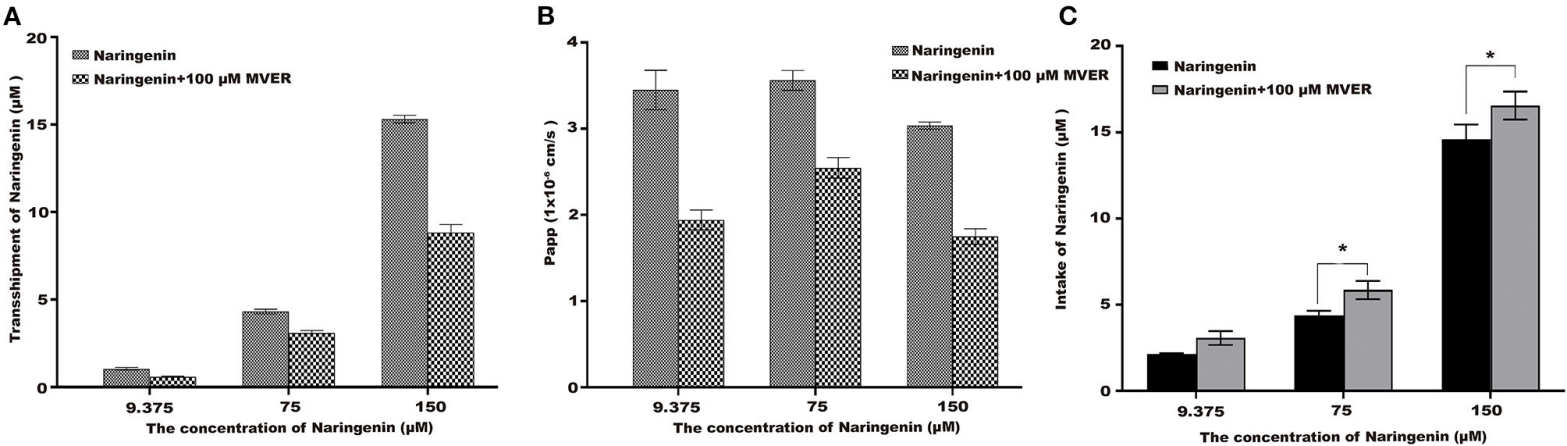
The effect of Verapamil (MVER) on naringenin transport and uptake is in Coca-2 cells. **(A)** The transport of different concentrations of naringenin in 120 min. **(B)** The Papp of each concentrations of naringenin during 120 min. **(C)** The intake of different concentrations of naringenin in 120 min. *p < 0.05 compared with Naringenin+Verapamil group.

To demonstrate the potential role of P-glycoprotein in the transport and uptake of naringenin across the Caco-2 cell monolayer, verapamil and ABCB1 shRNA were used to interfere with the transport and uptake of naringenin by Caco-2 (25, 26). After the application in the apical chamber prior to naringenin administration, Verapamil significantly lowered the transport of naringenin (Figures 7A,B). Compared with the inhibitor (100 μM Verapamil) group, Caco-2's intake of naringenin was significantly reduced in the control group (Figure 7C). The results showed that the transport of naringenin by Caco-2 cells depended on P-glycoprotein.

### Naringenin Protects the Cell Viability of H_2_O_2_-Stimulated Caco-2 Cells

**Figure 8:**
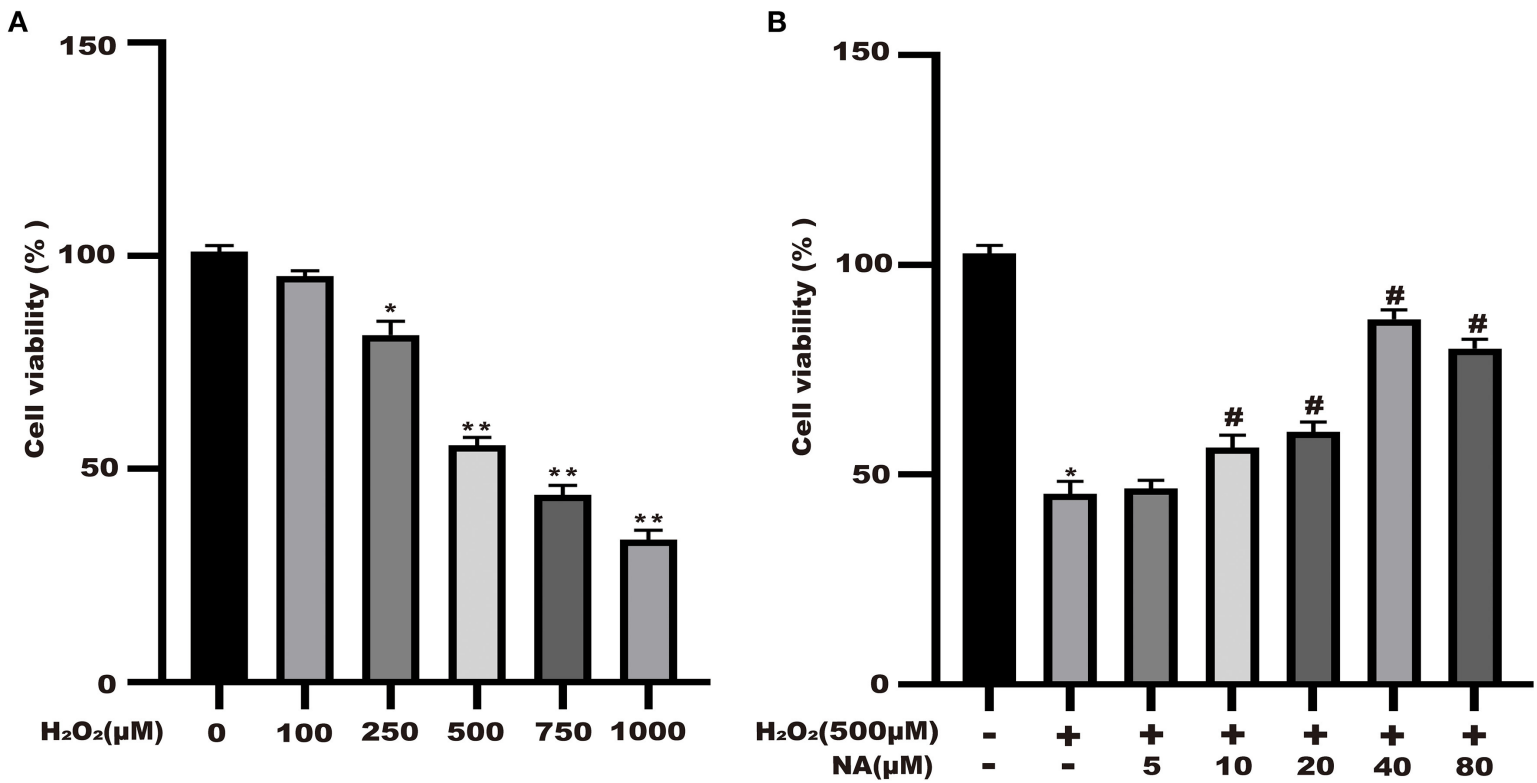
Naringenin protects the cell viability of H_2_O_2_-stimulated Caco-2. **(A)** H_2_O_2_ decreased the cell viability of Caco-2 cells in a dose-dependent manner. **(B)** Naringenin enhanced the cell viability of H_2_O_2_-induced Caco-2 cells. *p < 0.05 and **p < 0.05 compared with control group; ^#^*p* < 0.05 compared with H_2_O_2_ group.

Naringenin (9.375, 18.75, 37.5, 75, and 150 μM) had no significant effect on the activity of Caco-2 cells at 24 h in Figure 1. As shown in Figure 8A, the cell viability significantly decreased. The cell viability was reduced at 48.6% when 500 μM H_2_O_2_ was added for 3 h in the medium compared with the control group (Figure 8A). As shown in Figure 1B, when Caco-2 cells were pre-treated with different concentrations of naringenin for 24 h and then incubated with with 500 μM H_2_O_2_ for 3 h, naringenin showed a dose-dependent recovery of the cell viability. Therefore, 150 μM of naringenin was used in the subsequent experiments (Figure 8B).

### Metabolomics Analysis of Naringenin Effect on H_2_O_2_-Induced Caco-2 Cells

#### Metabolomics Analysis of Caco-2 Cells

**Figure 9:**
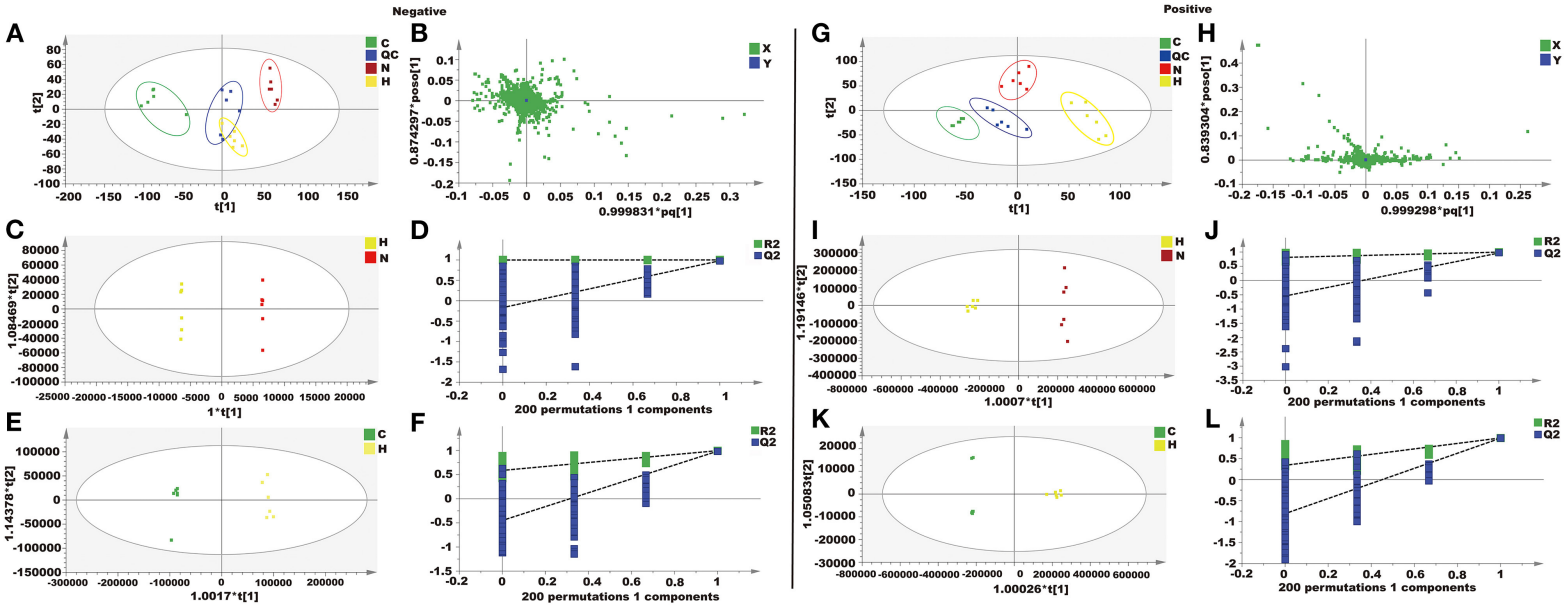
Metabolomics analysis of the effect of naringenin on H_2_O_2_-induced oxidative injury in Coca-2 cell. **(A,G)** PCA score plots. **(B,H)** The loading plot. **(C,I)** OPLS-DA score plots. **(D,J)** Permutation test of the OPLS-DA model. **(E,K)** OPLS-DA score plots. **(F,L)** Permutation test of the OPLS-DA model.

In this study, unsupervised PCA was performed on four groups of data (control group, H_2_O_2_ group, naringenin group, and QC group). The four groups showed clear separation in both positive and negative ion modes in the PCA plots (Figures 9A,B,G,H). The OPLS-DA model was constructed to further investigate and analyze the separation of the H_2_O_2_ group and other groups (control group and naringenin group). The results showed a clear separation of the H_2_O_2_ group from the other groups in the positive or negative mode in the OPLS-DA score plot (Figures 9C,E,I,K). The values of R2X, R2Y, and Q2 indicated that the model was stable and had good predictive power (Figures 9D,F,J,L).

Twenty four metabolites were identified as differential metabolites in Table 1, including sulfonic acid, pantothenic acid, methyltyrosine, acetyl-L-aspartic, glucuronic acid, cysteic acid, taurine, L-Histidine, L-glutamic acid, lactic acid, oxoproline, inosine, hypoxanthine, histidine, guanosine, guanine, pantothenic acid, citramalic acid. After pre-treatment with naringenin, the levels of these differential metabolites were normalized due to up- or down-regulation.

**Table 1:** Differential metabolites in Caco-2 cell.

**No**	**RT**	**VIP**	**Formual**	**Metabolites**	**SM**	**m/z**	**Fold change**	
							**C/H**	**N/H**
1	1.881573	2.18	C_5_H_4_N_4_O_2_	Xanthine	ESI+	151.0254	0.32	1.91*
2	1.832831	3.31	C_3_H_9_O_6_P	Glycerol 3-phosphate	ESI+	171.0078	1.88	2.17*
3	8.151354	1.36	C_17_H_20_N_4_O6	Riboflavin	ESI+	375.1308	1.11	2.17*
4	2.945925	1.23	C_5_H_7_NO_3_	Pyroglutamic acid	ESI+	128.0343	0.89	0.25*
5	1.696262	2.19	C_20_H_16_O_9_	Pyrogallin	ESI+	203.0369	1.09	3.27*
6	4.815042	2.21	C_4_H_11_O_4_P	Phosphoric acid	ESI+	96.96839	0.88	2.56*
7	3.336667	1.09	C_8_H_18_O_7_S	Sulfonic acid	ESI+	273.0383	0.41	1.16*
8	3.337222	2.11	C_9_H_17_NO_5_	Pantothenic acid	ESI+	218.1032	0.55	2.17*
9	14.40785	2.97	C_9_H_11_NO_2_	Methyltyrosine	ESI+	194.0817	0.45	1.66*
10	3.324992	5.21	C_14_H_14_N_2_O_5_	Acetyl-L-aspartic acid	ESI+	174.0397	2.18	0.86*
11	1.38885	3.21	C_6_H_10_O_7_	Glucuronic acid	ESI+	193.0349	0.65	1.92*
12	10.86779	1.99	C_3_H_7_NO_5_S	Cysteic acid	ESI+	167.9959	0.58	1.85*
13	3.515183	1.09	NH_2_CH_2_CH_2_SO_3_H	Taurine	ESI+	124.0062	1.92	0.57*
14	1.36195	2.51	C_6_H_9_N_3_O_2_	L-Histidine	ESI+	154.0616	0.77	1.68*
15	3.1647	2.51	C_5_H_9_NO_4_	L-Glutamic acid	ESI+	146.0449	0.68	0.18*
16	1.214575	3.88	C_3_H_6_O_3_	Lactic acid	ESI+	89.02312	0.27	1.72*
17	1.634986	1.71	C_5_H_7_NO_3_	Oxoproline	ESI+	128.0344	0.82	2.13*
18	1.53901	1.41	C_10_H_12_N_4_O_5_	Inosine	ESI+	267.0735	0.78	1.89*
19	1.538802	3.77	C_5_H_4_N_4_O	Hypoxanthine	ESI-	135.0304	0.77	1.81*
20	1.319585	5.06	C_6_H_9_N_3_O_2_	Histidine	ESI-	154.0615	0.77	2.78*
21	1.888793	1.17	C_10_H_13_N_5_O_5_	Guanosine	ESI-	282.0847	3.27	0.98*
22	1.633602	2.86	C_5_H_5_N_5_O	Guanine	ESI-	150.0412	0.67	2.66*
23	2.889838	3.90	C_9_H_17_NO_5_	Pantothenic acid	ESI-	218.1031	1.27	2.97*
24	1.885903	2.18	C_5_H_8_O_5_	Citramalic acid	ESI-	147.0291	0.78	1.89*

The * symbol indicates the value of p <0.05 compared with the H_2_O_2_ group.

#### Metabolic Pathway Analysis

**Figure 10:**
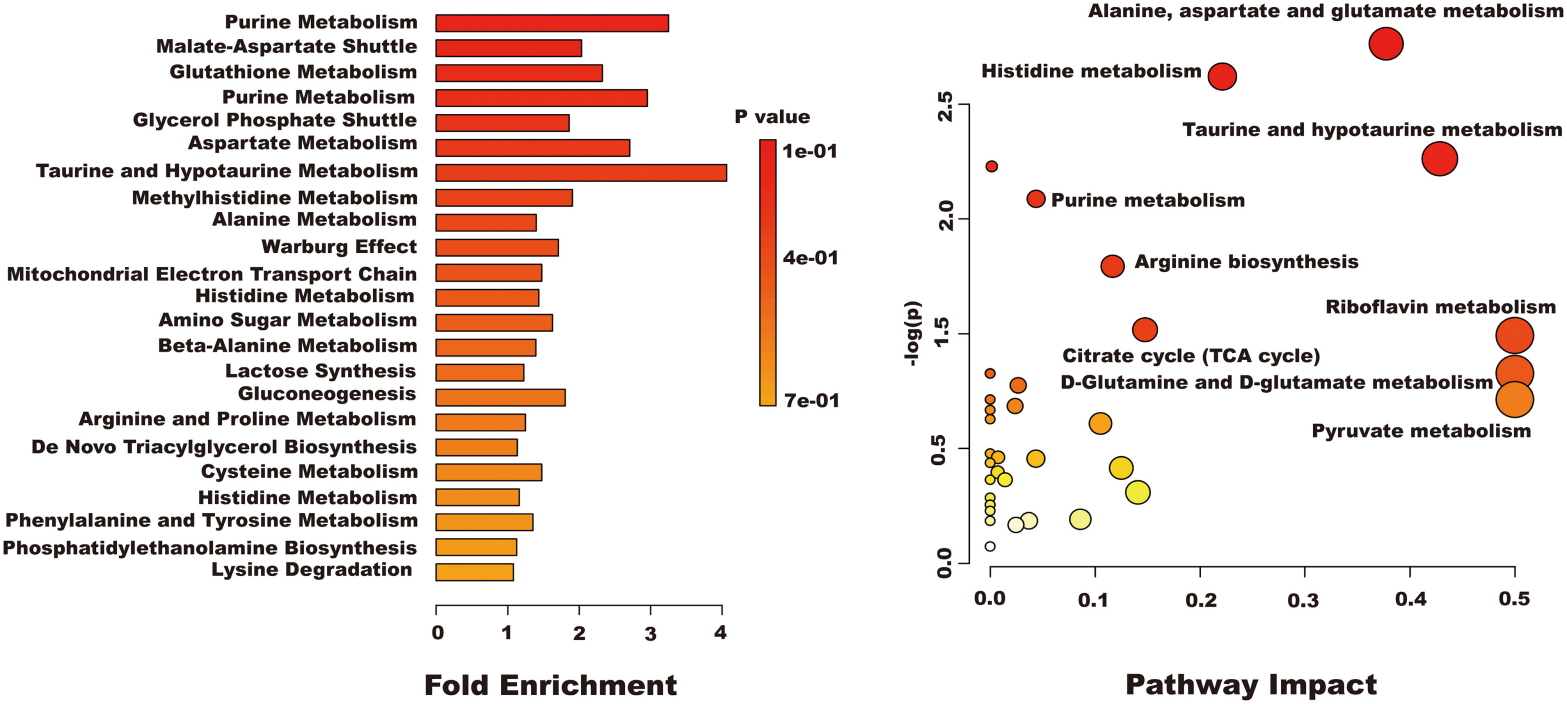
The results of enrichment and path impact of differential metabolites in Caco-2 cell.

**Figure 11:**
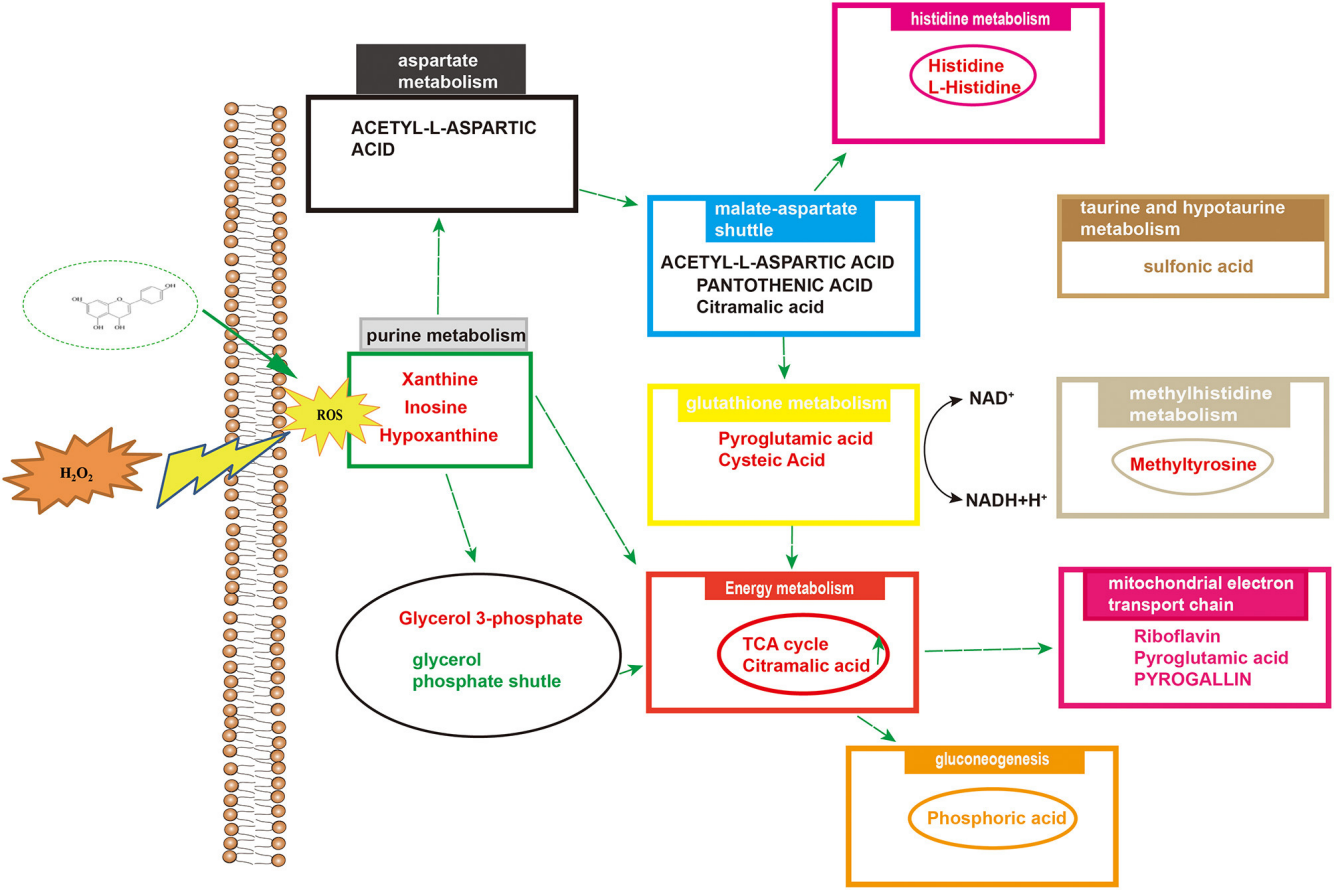
Overview of altered concentrations of metabolites and related metabolic pathways.

Differential metabolites were imported into MetaboAnalyst 5.0 for relevant metabolic pathway analysis and KEGG enrichment analysis. There are 19 main metabolic pathways: purine metabolism, malate-aspartate shuttle, glutathione metabolism, glycerol phosphate shutle, aspartate metabolism, taurine and hypotaurine metabolism, methylhistidine metabolism, alanine metabolism, warburg effect, mitochondrial electron transport chain, amino sugar metabolism, beta-alanine metabolism, lactose synthesis, gluconeogenesis, arginine and proline metabolism, *de novo* triacylglycerol biosynthesis, cysteine metabolism, histidine metabolism, lysine degradation (Figure 10). The influence of the path is mainly concentrated in alanine, aspartate and glutamate metabolism, histidine metabolism, taurine and hypotaurine metabolism, pyruvate metabolism, purine metabolism, arginine biosynthesis, citrate cycle, riboflavin metabolism, and D-glutamine and D-glutamate metabolism. H_2_O_2_-induced Caco-2 cells are mainly reflected in redox reactions, amino acid synthesis and metabolism, and energy metabolism (Figure 11). The results indicated that H_2_O_2_-induced oxidative damage could cause metabolic disturbances in Caco-2 cells, and naringenin pretreatment could effectively regulate this imbalance.

## Discussion

After oral administration of flavonoids in plant foods, their biological effects may be much weaker than *in vitro* studies (27, 28). In the past 20 years, this conclusion has been accepted by researchers. The pharmacokinetic characteristics have also been elucidated, including absorption, metabolism, disposal and elimination (29). In addition, studies have shown that flavonoids can only be absorbed by the body after the glycoside portion has been removed (30, 31). However, the transport and uptake of naringenin in this study could be observed in the Caco-2 cell model. The study showed that the transport of naringenin can be mediated by P-glycoprotein. However, some studies also have shown that the transport of naringenin does not depend on P-glycoprotein, but on the entry of MRP1 carriers into cells (32). In this study, naringenin absorption was polarized with Papp_BA_ superior to Papp_AB_, and naringenin is transported by active efflux protein carriers. Verapamil could significantly inhibit the transport of naringenin, and it showed that naringenin transport was most likely dependent on P-glycoprotein. Previous research had shown that naringenin can inhibit P-glycoprotein mediated efflux of vincristine in the blood-brain barrier (33, 34). Moreover, naringenin inhibit drug efflux by directly interacting with various sites of P-glycoprotein (35, 36).

The results of this experiment indicated that the Papp of naringenin in the Caco-2 cell monolayer was between 1.0 × 10^−6^ to 1.0 × 10^−5^ cm•s^−1^. This result can well predict the absorption mechanism of naringenin in the gut. If the macromolecule requires a paracellular pathway, the gap between cells can be opened (37). In this study, TEER did not decrease significantly, indicating that naringenin rarely crosses Caco-2 cells via paracellular pathways.

Flavonoids are a large group of plant-derived compounds, including quercetin and naringenin (38, 39). Quercetin and naringenin have similar physical and chemical properties. They are weakly acidic and have low water solubility. There have been many studies on the transport and absorption of quercetin. Studies have shown that quercetin glycosides might be actively absorbed in the human intestine via unspecified hexose transporters (40). The results confirmed the transport of naringenin in modeled Caco-2 cells and support the involvement of P-glycoprotein in this process.

A metabolomic approach based on LC-MS technology is a useful technique for evaluating the production of metabolites in cells under oxidative stress. Studies have shown that there are many metabolites of naringenin including apigenin, hesperetin, hippuric acid, 4-hydroxybenzoic acid and 3-(4′-hydroxyphenyl) propionic acid. These polyphenolic compounds play important roles in antioxidant, anti-inflammatory and anti-apoptotic roles (41, 42). In this study, we identified some metabolites of naringenin, such as apigenin and hesperetin. Apigenin has anti-inflammatory, antioxidant and anticancer properties. As a natural compound, apigenin may be an ideal and safe antitumor agent. Studies have shown that apigenin has good antitumor activity both *in vitro* and *in vivo* (43, 44). Hesperetin, a member of the flavonoid flavonoids, has been extensively studied for its anticancer, antioxidant, and anti-inflammatory properties. Hesperetin blocks neuroinflammation in microglia by regulating the expression of proteins associated with oxidative stress, inflammatory responses and apoptosis. In the present study, compared with control group, 24 differential metabolites were identified in Caco-2 cells pretreated with naringenin in the absence of H_2_O_2_ compared to the control group. These differential metabolites are involved in amino acid synthesis and metabolism, redox reactions, energy metabolism and cofactor metabolism. The levels of xanthine and hypoxanthine were significantly increased in the H_2_O_2_ group. The increased purines (xanthine and hypoxanthine) are often used as markers of oxidative stress. However, in this case, it is also possible that they represent loss of ATP as part of tissue loss and turnover (45, 46). The production of lactate and the reversible conversion of dihydroxyacetone phosphate to glycerol 3-phosphate catalyzed by glycerol-3-phosphate dehydrogenase involve the redox reactions of NADH and NAD^+^ (47, 48). After pretreating Caco-2 cells with naringenin, the levels of riboflavin were also markedly increased. As a natural antioxidant, riboflavin protects the body from oxidative stress (49). The levels of pyroglutamic acid and glutamic acid were significantly increased in the H_2_O_2_ group. Naringenin significantly reduced the elevation of sulfonic acid levels in Caco-2 cells. Studies have shown that under conditions of oxidative stress, H_2_O_2_ causes irreversible sulfinic and sulfonic acid modifications, which often lead to inactivation of antioxidant enzymes (50). Research has shown that rats deficient in pantothenic acid exhibit duodenitis and duodenal ulcers (51). N-acetyl-L-aspartic acid, cysteine and taurine are also good antioxidants (52). Hypoxanthine have been defined as biomarkers of hypoxia, hypoxemia, and ischemic brain injury (53, 54). Inosine is a natural analog of adenosine and binds adenosine receptors A3 (55, 56), thus decelerating inflammation. Histidine is an essential amino acid, and histidine supplementation inhibits inflammatory processes (57). Naringenin significantly increased histidine content in Caco-2 cells. Furthermore, *in vitro* experiments, histidine supplementation reduced the expression of IL-6 and TNF-α in adipocytes (58). Cellular metabolomics suggested that the underlying mechanism of naringenin's anti-oxidative stress effect may be related to increased anti-oxidative stress activity and decreased inflammatory response by improving metabolic changes.

## Conclusion

Naringenin can penetrate Caco-2 cells, mainly mediated by the active transport pathway involved in P-glycoprotein. By carefully examining the cellular absorption, and transport efficiency of naringenin and their antioxidant effect together, we can reasonably conclude that naringenin may play a more important role in preventing oxidative stress-induced intestinal diseases.

## Data Availability Statement

The original contributions presented in the study are included in the article/supplementary material, further inquiries can be directed to the corresponding author/s.

## Author Contributions

J-YL conceived and designed experiments. Z-DZ and QT performed the experiments and analyzed the data. X-WL synthesized and purified AEE. S-HL, Y-JY, ZQ, and L-XB supplied reagents. All authors contributed to the article and approved the submitted version.

## Funding

This study was supported by the Special Fund for National natural science foundation (Grant/Award Number: 31872518).

## Conflict of Interest

The authors declare that the research was conducted in the absence of any commercial or financial relationships that could be construed as a potential conflict of interest.

## Publisher's Note

All claims expressed in this article are solely those of the authors and do not necessarily represent those of their affiliated organizations, or those of the publisher, the editors and the reviewers. Any product that may be evaluated in this article, or claim that may be made by its manufacturer, is not guaranteed or endorsed by the publisher.

## References

[1] Cao Y Fan G Wang Z Gu Z. Phytoplasma-induced changes in the acetylome and succinylome of paulownia tomentosa provide evidence for involvement of acetylated proteins in witches' broom disease. Mol Cell Proteomics. (2019) 18:1210–26. 10.1074/mcp.RA118.001104 30936209 PMC6553929

[2] Streich K Smoczek M Hegermann J Dittrich-Breiholz O Bornemann M Siebert A. Dietary lipids accumulate in macrophages and stromal cells and change the microarchitecture of mesenteric lymph nodes. J Adv Res. (2020) 24:291–300. 10.1016/j.jare.2020.04.020 32405435 PMC7210474

[3] Steegenga WT de Wit NJ Boekschoten MV Ijssennagger N Lute C Keshtkar S. Structural, functional and molecular analysis of the effects of aging in the small intestine and colon of C57BL/6J mice. BMC Med Genomics. (2012) 5:38. 10.1186/1755-8794-5-38 22929163 PMC3534289

[4] Ducastel S Touche V Trabelsi MS Boulinguiez A Butruille L Nawrot M. The nuclear receptor FXR inhibits Glucagon-Like Peptide-1 secretion in response to microbiota-derived Short-Chain Fatty Acids. Sci Rep. (2020) 10:174. 10.1038/s41598-019-56743-x 31932631 PMC6957696

[5] Diukendjieva A Tsakovska I Alov P Pencheva T Pajeva I Worth AP. Advances in the prediction of gastrointestinal absorption:Quantitative Structure-Activity Relationship (QSAR) modelling of PAMPA permeability. Comput Toxicol. (2019) 10:51–9. 10.1016/j.comtox.2018.12.008

[6] Gerasimenko TN Senyavina NV Anisimov NU Tonevitskaya SA. A model of cadmium uptake and transport in Caco-2 cells bull. Exp Biol Med. (2016) 161:187–92. 10.1007/s10517-016-3373-7 27259497

[7] Pescio LG Favale NO Marquez MG Sterin-Speziale NB. Glycosphingolipid synthesis is essential for MDCK cell differentiation. Biochim Et Biophys Acta-Molec Cell Biol Lipids. (2012) 1821:884–94. 10.1016/j.bbalip.2012.02.009 22387616

[8] Lapczuk-Romanska J Wajda A Pius-Sadowska E Kurzawski M Niedzielski A Machalinski B. Effects of simvastatin on nuclear receptors, drug metabolizing enzymes and transporters expression in Human Umbilical Vein Endothelial Cells. Pharmacol Rep. (2018) 70:875–80. 10.1016/j.pharep.2018.03.008 30092417

[9] He X Sugawara M Kobayashi M Takekuma Y Miyazaki K. An in vitro system for prediction of oral absorption of relatively water-soluble drugs and ester prodrugs. Int J Pharm. (2003) 263:35–44. 10.1016/S0378-5173(03)00343-0 12954178

[10] Fujikawa M Ano R Nakao K Shimizu R Akamatsu M. Relationships between structure and high-throughput screening permeability of diverse drugs with artificial membranes: application to prediction of Caco-2 cell permeability. Bioorg Med Chem. (2005) 13:4721–32. 10.1016/j.bmc.2005.04.076 15936203

[11] Artursson P Palm K Luthman K. Caco-2 monolayers in experimental and theoretical predictions of drug transport. Adv Drug Deliv Rev. (2012) 64:280–9. 10.1016/j.addr.2012.09.005 11259831

[12] Ullah A Munir S Badshah SL Khan N Ghani L Poulson BG. Important flavonoids and their role as a therapeutic agent. Molecules. (2020) 25:5243. 10.3390/molecules25225243 33187049 PMC7697716

[13] Subramanya SB Venkataraman B Meeran MF Goyal SN Patil CR Ojha S. Therapeutic potential of plants and plant derived phytochemicals against acetaminophen-induced liver injury. Int J Molec Sci. (2018) 19:3776. 10.3390/ijms19123776 30486484 PMC6321362

[14] Li S Zhang Y Sun Y Zhang G Bai J Guo J. Naringenin improves insulin sensitivity in gestational diabetes mellitus mice through AMPK. Nutr Diab. (2019) 9:1–10. 10.1038/s41387-019-0095-8 31591391 PMC6779739

[15] Bodet C La VD Epifano F Grenier D. Naringenin has anti-inflammatory properties in macrophage and ex vivo human whole-blood models. J Periodontal Res. (2008) 43:400–7. 10.1111/j.1600-0765.2007.01055.x 18503517

[16] Meng LM Ma HJ Guo H Kong QQ Zhang Y. The cardioprotective effect of naringenin against ischemia-reperfusion injury through activation of ATP-sensitive potassium channel in rat. Can J Physiol Pharmacol. (2016) 94:973–8. 10.1139/cjpp-2016-0008 27408985

[17] Mulvihill EE Allister EM Sutherland BG Telford DE Sawyez CG Edwards JY. Naringenin prevents dyslipidemia, apolipoprotein B overproduction, and hyperinsulinemia in LDL receptor-null mice with diet-induced insulin resistance. Diabetes. (2009) 58:2198–210. 10.2337/db09-0634 19592617 PMC2750228

[18] Kawser Hossain M Abdal Dayem A Han J Yin Y Kim K Kumar Saha S. Molecular mechanisms of the anti-obesity and anti-diabetic properties of flavonoids. Int J Molec Sci. (2016) 17:569. 10.3390/ijms17040569 27092490 PMC4849025

[19] Tsai SJ Huang CS Mong MC Kam WY Huang HY Yin MC. Anti-inflammatory and antifibrotic effects of naringenin in diabetic mice. J Agric Food Chem. (2012) 60:514–21. 10.1021/jf203259h 22117528

[20] Al-Ghamdi N Virk P Hendi A Awad M Mai E. Antioxidant potential of bulk and nanoparticles of naringenin against cadmium-induced oxidative stress in Nile tilapia, Oreochromis niloticus. Green Process Synth. (2021) 10:392–402. 10.1515/gps-2021-0037

[21] Zhao Q Yang HY Liu F Luo JY Zhao Q Li XM. Naringenin exerts cardiovascular protective effect in a palmitate-induced human umbilical vein endothelial cell injury model via autophagy flux improvement. Mol Nutr Food Res. (2019). 10.1002/mnfr.201900601 31622021

[22] Huang MZ Yang YJ Liu XW Qin Z Li JY. Aspirin eugenol ester reduces H2O2-induced oxidative stress of HUVECs via mitochondria-lysosome axis. Oxid Med Cell Longev. (2019) 2019:8098135. 10.1155/2019/8098135 31583045 PMC6754946

[23] Xiang Q Zhang W Li Q Zhao J Feng W Zhao T. Investigation of the uptake and transport of polysaccharide from Se-enriched Grifola frondosa in Caco-2 cells model. Int J Biol Macromol. (2020) 158:1330–41. 10.1016/j.ijbiomac.2020.04.160 32339585

[24] Zeng Z Shen ZL Zhai S Xu JL Liang H Shen Q. Transport of curcumin derivatives in Caco-2 cell monolayers. Eur J Pharm Biopharm. (2017) 117:123–31. 10.1016/j.ejpb.2017.04.004 28396278

[25] Xu Q Hong H Wu J Yan X. Bioavailability of bioactive peptides derived from food proteins across the intestinal epithelial membrane: a review. Trends Food Sci Technol. (2019) 86:399–411. 10.1016/j.tifs.2019.02.050

[26] Xu Q Fan H Yu W Hong H Wu J. Transport study of egg-derived antihypertensive peptides (LKP and IQW) using Caco-2 and HT29 coculture monolayers. J Agric Food Chem. (2017) 65:7406–14. 10.1021/acs.jafc.7b02176 28782363

[27] Justino GC Santos MR Canario S Borges C Florencio MH Mira L. Plasma quercetin metabolites: structure-antioxidant activity relationships. Arch Biochem Biophys. (2004) 432:109–21. 10.1016/j.abb.2004.09.007 15519302

[28] Santos MR Rodriguez-Gomez MJ Justino GC Charro N Florencio MH Mira L. Influence of the metabolic profile on the in vivo antioxidant activity of quercetin under a low dosage oral regimen in rats. Br J Pharmacol. (2008) 153:1750–61. 10.1038/bjp.2008.46 18311191 PMC2438264

[29] Gonzales GB Smagghe G Grootaert C Zotti M Raes K Van Camp J. Flavonoid interactions during digestion, absorption, distribution and metabolism:a sequential structure-activity/property relationship-based approach in the study of bioavailability and bioactivity. Drug Metab Rev. (2015) 47:175–90. 10.3109/03602532.2014.1003649 25633078

[30] Tronina T Strugała P Popłoński J Włoch A Sordon S Bartmańska A. The influence of glycosylation of natural and synthetic prenylated flavonoids on binding to human serum albumin and inhibition of cyclooxygenases COX-1 and COX-2. Molecules. (2017) 22:1230. 10.3390/molecules22071230 28754033 PMC6152009

[31] Fernando W Goralski KB Hoskin DW Rupasinghe HPV. Metabolism and pharmacokinetics of a novel polyphenol fatty acid ester phloridzin docosahexaenoate in Balb/c female mice. Sci Rep. (2020) 10:21391. 10.1038/s41598-020-78369-0 33288802 PMC7721897

[32] Nait Chabane M Al Ahmad A Peluso J Muller CD Ubeaud G. Quercetin and naringenin transport across human intestinal Caco-2 cells. J Pharm Pharmacol. (2009) 61:1473–83. 10.1211/jpp.61.11.0006 19903372

[33] Takanaga H Ohnishi A Matsuo H Sawada Y. Inhibition of vinblastine efflux mediated by P-glycoprotein by grapefruit juice components in caco-2 cells. Biol Pharm Bull. (1998) 21:1062–1066. 10.1248/bpb.21.1062 9821810

[34] Milanei HA Al Ahmad A Naitchabane M Vandamme TF Jung L Ubeaud G. Transport of quercetin di-sodium salt in the human intestinal epithelial Caco-2 cell monolayer 139. Eur J Drug Metab Pharmacokinet. (2007) 32:139–47. 10.1007/BF03190476 18062406

[35] Mitsunaga Y Takanaga H Matsuo H Naito M Tsuruo T Ohtani H. Effect of bioflavonoids on vincristine transport across blood-brain barrier. Eur J Pharmacol. (2000) 395:193–201. 10.1016/S0014-2999(00)00180-1 10812049

[36] Zhou SF Lim LY Chowbay B. Herbal modulation of P-glycoprotein. Drug Metab Rev. (2004) 36:57–104. 10.1081/DMR-120028427 15072439

[37] Fu QX Wang HZ Xia MX Deng B Shen HY Ji G. The effect of phytic acid on tight junctions in the human intestinal Caco-2 cell line and its mechanism. Eur J Pharmaceut Sci. (2015) 80:1–8. 10.1016/j.ejps.2015.09.009 26385515

[38] Gansukh E Gopal J Paul D Muthu M Kim DH Oh JW. Ultrasound mediated accelerated Anti-influenza activity of Aloe vera. Sci Rep. (2018) 8:1–10. 10.1038/s41598-018-35935-x 30542141 PMC6290770

[39] Lee SB Kang JW Kim SJ Ahn J Kim J Lee SM. Afzelin ameliorates D-galactosamine and lipopolysaccharide-induced fulminant hepatic failure by modulating mitochondrial quality control and dynamics. Br J Pharmacol. (2017) 174:195–209. 10.1111/bph.13669 27861739 PMC5192940

[40] Walters HC Craddock AL Fusegawa H Willingham MC Dawson PA. Expression, transport properties, and chromosomal location of organic anion transporter subtype 3. Am J Physiol Gastrointest Liver Physiol. (2000) 279:G1188–1200. 10.1152/ajpgi.2000.279.6.G1188 11093941

[41] Tan J Li Y Hou DX Wu S. The effects and mechanisms of cyanidin-3-glucoside and its phenolic metabolites in maintaining intestinal integrity. Antioxidants. (2019) 8. 10.3390/antiox8100479 PMC6826635 31614770

[42] Hu R Wu S Li B Tan J Yan J Wang Y. Dietary ferulic acid and vanillic acid on inflammation, gut barrier function and growth performance in lipopolysaccharide-challenged piglets. Anim Nutr. (2022) 8:144–52. 10.1016/j.aninu.2021.06.009 34977384 PMC8683658

[43] Wang QR Yao XQ Wen G Fan Q Li YJ Fu XQ. Apigenin suppresses the growth of colorectal cancer xenografts via phosphorylation and up-regulated FADD expression. Oncol Lett. (2011) 2:43–7. 10.3892/ol.2010.215 22870126 PMC3412520

[44] Hu XW Meng D Fang J. Apigenin inhibited migration and invasion of human ovarian cancer A2780 cells through focal adhesion kinase. Carcinogenesis. (2008) 29:2369–76. 10.1093/carcin/bgn244 18974065

[45] Quinlan GJ Lamb NJ Tilley R Evans TW Gutteridge JM. Plasma hypoxanthine levels in ARDS:implications for oxidative stress, morbidity, and mortality. Am J Respir Crit Care Med. (1997) 155:479–484. 10.1164/ajrccm.155.2.9032182 9032182

[46] Marrocco I Altieri F Peluso I. Measurement and clinical significance of biomarkers of oxidative stress in humans. Oxid Med Cell Longev. (2017) 2017:6501046. 10.1155/2017/6501046 28698768 PMC5494111

[47] Vander Heiden MG Cantley LC Thompson CB. Understanding the Warburg effect:the metabolic requirements of cell proliferation. Science. (2009) 324:1029–33. 10.1126/science.1160809 19460998 PMC2849637

[48] Feng X Tjia J Zhou Y Liu Q Yang H. Effects of tocopherol nanoemulsion addition on fish sausage properties and fatty acid oxidation. LWT- Food Sci Technol. (2020) 118:108737. 10.1016/j.lwt.2019.108737

[49] Ashoori M Saedisomeolia A. Riboflavin (vitamin B(2)) and oxidative stress:a review. Br J Nutr. (2014) 111:1985–91. 10.1017/S0007114514000178 24650639

[50] Riemer J Schwarzlander M Conrad M Herrmann JM. Thiol switches in mitochondria:operation and physiological relevance. Biol Chem. (2015) 396:465–82. 10.1515/hsz-2014-0293 25720067

[51] Berg BN. Duodenitis and duodenal ulcers produced in rats by pantothenic acid deficiency. Br J Exp Pathol. (1959) 40:371–374. 13799206 PMC2082259

[52] Grove RQ Karpowicz SJ. Reaction of hypotaurine or taurine with superoxide produces the organic peroxysulfonic acid peroxytaurine. Free Radic Biol Med. (2017) 108:575–84. 10.1016/j.freeradbiomed.2017.04.342 28438660

[53] Lewis GD Wei R Liu E Yang E Shi X Martinovic M. Metabolite profiling of blood from individuals undergoing planned myocardial infarction reveals early markers of myocardial injury. J Clin Invest. (2008) 118:3503–12. 10.1172/JCI35111 18769631 PMC2525696

[54] Bell MJ Kochanek PM Carcillo JA Mi Z Schiding JK Wisniewski SR. Interstitial adenosine, inosine, and hypoxanthine are increased after experimental traumatic brain injury in the rat. J Neurotrauma. (1998) 15:163–170. 10.1089/neu.1998.15.163 9528916

[55] Jin X Shepherd RK Duling BR Linden J. Inosine binds to A3 adenosine receptors and stimulates mast cell degranulation. J Clin Invest. (1997) 100:2849–2857. 10.1172/JCI119833 9389751 PMC508491

[56] Gomez G Sitkovsky MV. Differential requirement for A2a and A3 adenosine receptors for the protective effect of inosine in vivo. Blood. (2003) 102:4472–8. 10.1182/blood-2002-11-3624 12947007

[57] Young VR. Adult amino acid requirements:the case for a major revision in current recommendations. J Nutr. (1994) 124:1517S−1523S. 10.1093/jn/124.suppl_8.1517S 8064412

[58] Feng RN Niu YC Sun XW Li Q Zhao C Wang C. Histidine supplementation improves insulin resistance through suppressed inflammation in obese women with the metabolic syndrome: a randomised controlled trial. Diabetologia. (2013) 56:985–94. 10.1007/s00125-013-2839-7 23361591

